# Paramedic attitudes and experiences working as a community paramedic: a qualitative survey

**DOI:** 10.1186/s12873-024-00972-5

**Published:** 2024-04-01

**Authors:** Aarani Paramalingam, Andrea Ziesmann, Melissa Pirrie, Francine Marzanek, Ricardo Angeles, Gina Agarwal

**Affiliations:** 1 Department of Family Medicine, McMaster University, 100 Main St W, Hamilton, ON L8P 1H6 Canada; 2 Department of Health Research Methods, Evidence, and Impact, McMaster University, 1280 Main Street West, Hamilton, Ontario L8S 4L8 Canada

**Keywords:** Community Paramedicine, Paramedic, Professional identity, Mental health, Thematic analysis

## Abstract

**Background:**

Community paramedicine (CP) is an extension of the traditional paramedic role, where paramedics provide non-acute care to patients in non-emergent conditions. Due to its success in reducing burden on hospital systems and improving patient outcomes, this type of paramedic role is being increasingly implemented within communities and health systems across Ontario. Previous literature has focused on the patient experience with CP programs, but there is lack of research on the paramedic perspective in this role. This paper aims to understand the perspectives and experiences, both positive and negative, of paramedics working in a CP program towards the community paramedic role.

**Methods:**

An online survey was distributed through multiple communication channels (e.g. professional organizations, paramedic services, social media) and convenience sampling was used. Five open-ended questions asked paramedics about their perceptions and experiences with the CP role; the survey also collected demographic data. While the full survey was open to all paramedics, only those who had experience in a CP role were included in the current study. The data was qualitatively analyzed using a comparative thematic analysis.

**Results:**

Data was collected from 79 respondents who had worked in a CP program. Three overarching themes, with multiple sub-themes, were identified. The first theme was that CP programs fill important gaps in the healthcare system. The second was that they provide paramedics with an opportunity for lateral career movement in a role where they can have deeper patient connections. The third was that CP has created a paradigm shift within paramedicine, extending the traditional scope of the practice. While paramedics largely reported positive experiences, there were some negative perceptions regarding the slower pace of work and the “soft skills” required in the role that vary from the traditional paramedic identity.

**Conclusions:**

CP programs utilize paramedic skills to fill a gap in the healthcare system, can improve paramedic mental health, and also provide a new pathway for paramedic careers. As a new role, there are some challenges that CP program planners should take into consideration, such as additional training needs and the varying perceptions of CP.

**Supplementary Information:**

The online version contains supplementary material available at 10.1186/s12873-024-00972-5.

## Background

Community paramedicine (CP) is an emerging professional role where paramedics use their training and skills in emergency response to respond to individuals with non-acute needs who do not require transport to hospital [1]. In Ontario, Canada, CP programs have begun to garner attention as an innovative approach to support independent living in an aging older adult population with complex health conditions [2]. Although there were some very early adopters of CP programs in Ontario, these programs began to gain momentum in 2013 [3]. By 2014, 13 Paramedic Services in Ontario reported having CP programs [2]. Community paramedicine programs can be diverse in scope, and can include paramedics completing home visits to frequent 911 callers, supporting clients with healthcare navigation, providing community-based education, and conducting drop-in clinic style wellness programs [1]. The structure, mandate, and resources required for CP programs tend to vary by paramedic service and local contexts. Staffing and training arrangements can also vary, with some programs designating full-time ‘community paramedics’ while others deploy paramedics on modified duties to staff programs.

Our literature review found that few studies have sought to understand how paramedics experience and view these programs. Evaluations of CP tend to focus on patient experiences, such as their health outcomes and health service utilization [4–6]. While participants have generally expressed support for and acceptance of CP [5, 6], it is unclear exactly how paramedics perceive CP programs, particularly as it relates to their understanding of paramedic professional identity and their mental health.

As the CP role becomes a more permanent part of paramedic practice, it is expected to redefine and broaden the paramedic identity beyond its traditional boundaries. Historically, service users and healthcare providers have defined paramedics as thrill seekers who provide transport, emergency response, and trauma care [7]. However, as the delivery of healthcare has become more complex and integrated, paramedic identity has also shifted. Paramedics in Canada have already adopted broad professional identities such as ‘clinician,’ ‘educator,’ ‘team member,’ and ‘patient advocate’ [8]. This expansion of the paramedic identity is expected to accelerate as CP programs are increasingly adopted in Ontario. CP programs require paramedics to work with individuals on a repeat basis, provide chronic disease management services, and use ‘soft’ skills such as motivational interviewing and advocacy. How paramedics feel about these changes to their professional identity as a result of CP has yet to be understood.

Additionally, participation in the CP role may alter paramedics’ mental health experience. Paramedics in traditional emergency response roles tend to experience Occupational Stress Injury (OSI) due to demanding work environments and exposure to traumatic incidents [9, 10]. Occupational Stress Injury refers to any form of psychological stress resulting from the duties one performs on the job [9]. While OSI is common for all public safety personnel, some studies suggest a higher incidence of post traumatic stress disorder for paramedics when compared to police officers and firefighters [11, 12]. Paramedics are estimated to be at higher risk of screening positive for a DSM-IV mental disorder than municipal or provincial police services, firefighters, and dispatchers [12]. While some preliminary research in one CP program suggests that paramedics who practice CP experience reduced stress and a greater quality of work life [9], it is unclear how working in CP programs in different capacities may alter paramedics’ exposure to OSI and affect one’s overall mental health.

This paper seeks to describe the positive and negative experiences of paramedics working in a CP program and assess CP’s impacts on paramedic professional identity and paramedic’s mental health experience. As paramedic experiences may not be aligned with the experiences of CP program participants or even paramedic leadership, this paper also seeks to identify workplace elements (e.g., training, supports, paramedic leadership and culture) that may promote or hinder the expansion of CP programs in Ontario.

## Methods

### Design

A survey tool was developed and distributed by the McMaster Community Paramedicine Research Team in 2016, using the online platform FluidSurveys, to assess paramedics’ perceptions and experiences working in a CP role. The survey was developed based on recurring themes and insights from a focus group and three key informant interviews with paramedics. The survey drafts were also reviewed and approved by a paramedic and a paramedic superintendent with research experience. The survey tool used open-ended questions to have paramedics describe their perception of the CP role prior to, and after working in a CP program, including both positive and negative aspects.

### Population and recruitment

Paramedics were invited to participate in a survey that was distributed through social media by the Ontario Paramedic Association and the CP@clinic program. On Twitter, the invitation to complete the survey was re-tweeted by multiple accounts including paramedic services, paramedic staff, and other accounts. In addition, some Paramedic Services in Ontario delivering CP programs emailed the survey link to their paramedic staff. All paramedics (with and without CP experience) were invited to complete the full survey, but only those who indicated that they had worked in a CP role were included in this study (screening question in the survey). Respondents were informed about the purpose of the research study and informed consent was obtained. This study was approved by the Hamilton Integrated Research Ethics Board (Project #13-466).

### Data collection

A convenience sample was collected using an online survey. The survey was available for 16 weeks from October 2016 to January 2017, to provide ample time to gather responses from all potential participants. Data from the open-ended questions were collated into a single transcript.

The survey collected the following demographic information: age, sex, years of service, type of paramedic training (i.e., primary care, advanced care, critical care), whether the paramedic was on modified duty while working in a CP program (i.e., awaiting return to regular duties), length of time working in CP programs, and types of programs they worked in. Fivetypes of CP programs were provided as options: home visit program, clinic style program, paramedic navigator style program, triage program, and other.

The following open-ended questions were asked to elicit responses about paramedics’ experience of the CP role:

1. What was your opinion of community paramedicine before working a community paramedicine role?
2. Please explain how your opinion of community paramedicine has changed since working in a community paramedic role?
3. What was positive about your experience working in a community paramedic role? What did you enjoy about this role?
4. What were the negative aspects in your experience working as a community paramedic?
5. Would you like to change anything about the community paramedic role?

### Analysis

A comparative thematic analysis was used to describe the experiences of community paramedics before and after working in a CP role. Two members of the research team (AP, AZ) independently coded responses and identified emergent themes. Using a phenomenological approach during secondary coding, coders grounded the emergent themes within paramedics’ lived experience of the community paramedicine role, finding explanations for their experience within the context of the data itself. Responses with thick narrative descriptions were retained for analysis. Incomplete or partial responses were included in the qualitative analysis. Themes were then synthesized, refined, and were validated and triangulated by research team members (GA, AZ, MP, FM, RA). The demographic data was analyzed using descriptive analysis.

## Results

### Demographics

Of the total survey respondents (*n*=434), 79 reported working in a CP role. These respondents were predominantly male (57.0%), had 10 or more years of experience in a paramedic role (77.2%), and were not on modified duty while working in a CP role (86.1%). Respondents reported experience with working in multiple types of CP programs, with the most common type being clinic style programs (68.4%) (see Table 1). While the survey was open to all paramedics, the majority of respondents report working in Ontario (*n*=61, 77.2%) and 16 respondents (20.3%) did not provide the province in which they worked.

**Table 1:** Demographics of survey respondents (*n*=79)

		**n (%)**
Sex	Female	29 (36.7)
	Male	45 (57.0)
	Not given	5 (6.3)
Age	20-24 years old	5 (6.3)
	25-29 years old	5 (6.3)
	30-34 years old	9 (11.4)
	35-39 years old	16 (20.3)
	40-44 years old	11 (13.9)
	45-49 years old	11 (13.9)
	50+ years old	18 (22.8)
	Not given	4 (5.1)
Years of Service	0-4 years	7 (8.9)
	5-9 years	7 (8.9)
	10-14 years	26 (32.9)
	15-19 years	10 (12.7)
	20+ years	25 (31.6)
	Not given	4 (5.1)
Paramedic training	PCP	31 (39.2)
	ACP	33 (41.8)
	Critical care	1 (1.3)
	Other	10 (12.7)
	Not given	4 (5.1)
Modified when working in CP	Yes	8 (10.1)
	No	68 (86.1)
	Not given	3 (3.8)
Type of program they worked in (Check all that apply)	Home visit program	46 (58.2)
	Clinic style program	54 (68.4)
	Paramedic navigator style program	18 (22.8)
	Triage program	1 (1.3)
	Other^a^	24 (30.4)
Length of time working in CP	Less than 6 months	25 (31.6)
	6 months to less than 12 months	19 (24.1)
	12 months to less than 24 months	12 (15.2)
	24 months or longer	20 (25.3)
	Not given	3 (3.8)

^a^e.g. referral programs, remote patient monitoring

**Fig. 1:**
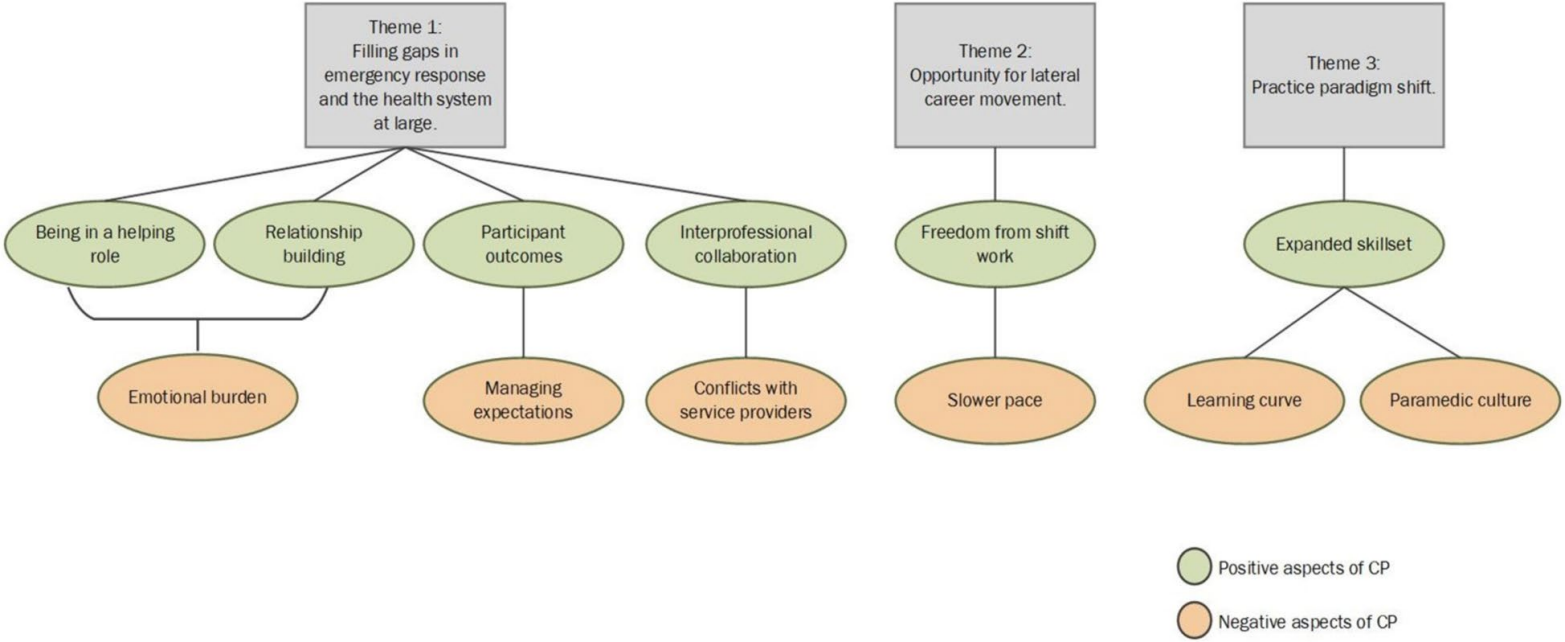
Diagram depicting the major themes and the positive and negative experiences of paramedics working in a CP role

A number of themes and sub-themes emerged from the analysis. Before having worked in a CP program, paramedics broadly identified three unique opportunities and impacts of the CP role: 1) filling gaps in emergency response and the healthcare system at large, 2) providing opportunity for lateral career movement, and 3) creating practice paradigm shifts. After working in a CP role, respondents were able to describe in detail the positive and negative aspects of these three opportunities and impacts. These themes are conceptualized in Fig. 1.

### Theme 1: CP programs can fill important gaps in emergency response and the healthcare system at large, but come with new professional challenges

Before working in a CP role, the majority of respondents viewed the CP role positively. CP was thought to fill important gaps in emergency response and the health system at large. It offered paramedics an opportunity to practice continuity of care by providing prevention and disease management support to older adults who were often inappropriately accessing emergency care services. Paramedics felt that the needs of these individuals were not being fulfilled through traditional emergency response.

*There are several individuals I have come across in my career who would have benefitted from a regularly scheduled home visit. ...There are a lot of individuals who require that [health] maintenance… it greatly reduces the workload of Emergency Services and frees them up for what they are actually required for – emergencies. (P.24)*

*[I thought] it was a vital service that filled gaps in the health care sector that was having excellent results where implemented (P.43)*

After working in a CP program, respondents expanded on these initial sentiments. They described delivering a different level of care to their communities that involved stepping into a novel helping role, building relationships with participants and their families, supporting participant health outcomes, and taking part in interprofessional collaboration. This new level of care also came with new professional challenges such as increased emotional burden, managing participant expectations, and conflicts with other health and social service providers.

#### Sub-theme 1A: being in a helping role

Helping program participants in a CP role was described as novel and different when compared to the emergency response role. Community paramedics worked with participants on a long-term basis and witnessed their health and quality of life improvements. Paramedics enjoyed helping participants who were part of vulnerable or underserved communities. By taking time to listen to these participants and hear their stories, paramedics were able to exercise more compassion and felt less judgemental about participants’ situations. This was a rewarding aspect of the CP role, even having a powerful positive effect on paramedics’ own mental health.

*Making a difference in people's lives ... often the people in the community who are ignored and shunned by others. I enjoyed going out in the community, solving problems, working with other services, having the time to LISTEN to patients rather than be worried about my scene time...this is one of the most important things for Paramedic mental health as well. (P.46)*

*...the knowledge that community paramedics, with sometimes very simple interventions/strategies can make all the difference in people's lives, preventing people from falling through the cracks, or helping them out of that situation…(P.61)*

#### Sub-theme 1B: relationship building with program participants

Paramedics enjoyed building relationships with participants and getting to know them on a personal level, which was not possible in an emergency response role due to limited time on scene during acute calls. Building rapport with participants in the comfort of their homes created a sense of trust that fostered into natural friendships, with some paramedics describing themselves as building a ‘family’ with participants. Others noted that this trust allowed participants to share more details about their health and medical history, allowing paramedics to better assist in their care. Paramedics felt it was important to build these strong social relationships with participants in order to encourage and affect health behaviour changes for participants. Strong relationships with participants allow paramedics to thoroughly follow-up after initial visits and engage in conversations about participants’ short- and long-term health goals. Additionally, although the CP role lacked the adrenaline rush, this increased socialization was described as filling this gap.

*The paramedics have built a rapport with [participants] and have really built a family with them.(P.19)*

*Getting to know [participants] beyond the 30 minutes to an hour we’re used to being with [them in an emergency capacity]. I found as they got to know me, they were more willing to share health concerns they were having and trusted me more. (P.26)*

*I realized that community paramedicine can be more enjoyable than I thought…where it lacks in adrenaline it makes up for in a social aspect. (P.10)*

*Seeing how much they trust us and tell us some of their most intimate issues. (P.49)*

#### Sub-theme 1C: emotional burden

While paramedics enjoyed the rapport and relationships built with participants, they also felt they were making greater emotional investments in participants who were in poor health, may have been in a palliative state or dealing with addictions issues. Burnout, attachment fatigue, and difficulty dealing with participant deaths were common experiences. For some paramedics, having built rapport with certain participants meant that they were the primary contact for follow-up care even on their days off, leading to poor work-life balance. Similar to other clinical practitioners who work one-on-one with individuals over a long period of time (e.g., physicians, social workers), one respondent emphasized the need for paramedics in a CP role to be trained to reflect on their experience and make adjustments to how they work with participants.

*Can be emotionally draining working over the long term with [participants]... who are very sick, some are palliative, difficult personalities, addictions, etc. Paramedics historically aren’t used to becoming emotionally involved with [people] … but this is difficult not to do when you are seeing people over and over again, and getting involved with their families and other circles of care as well. (P.5)*

*Couldn't just leave work behind at work like a traditional paramedic could - had to field phone calls on my vacation to help make arrangements for a [participant]... because no other community paramedics were available or as familiar with [them]. (P. 9)*

*Paramedics are not usually trained, educated, or encouraged to engage in self-reflective or reflective practice and it’s essential for a role like community paramedicine. (P. 34)*

#### Sub-theme 1D: participant outcomes

Paramedics reported a better understanding of the impact of CP programing on participants’ health and well-being. Identifying ‘silent’ health issues before they resulted in emergency transport, making appropriate referrals and reducing 911 calls were some of the positive outcomes. For some, their CP training had become an integral part of their role as a paramedic overall, providing valuable transferable skills that could also be used during an emergency response to further improve health outcomes and close gaps in care. Additionally, beyond identifying health issues and making appropriate referrals, some paramedics felt that CP programs help build a sense of community, which may in turn also improve participant health and well-being. Paramedics particularly appreciated being able to witness these positive outcomes first-hand.

*I have realized that community paramedicine has a very broad impact in the community. It is very underappreciated ... It has improved the livelihood of many [participants], and can (with the aid of other resources), assist them [with] their healthcare needs. (P.9)*

*Seeing them get proper treatment for an illness they did not know they had (i.e. hypertension, diabetes). (P.62)*

*Seeing the direct benefit of timely and appropriate interventions; having a big impact on people's quality of life, even when palliative (P. 60)*

*I see that most [people] don't want to go to the hospital and really don't need to. The issue is [that in] our current system people expect to be taken as they think that's the only way a doctor will see them. When they realised someone could see them at home and then refer them to the required service less 911 calls were made. (P.10)*

*I'm fortunate enough to work in a service that has integrated some aspects of community paramedicine into every response. Being trained to recognize signs in a [participant]'s home that indicate a higher need for home care and offering ways for them to access more care is deeply satisfying. The relief on a person's face when told they could get some home care, or help with day to day chores makes me feel like I made a difference to their quality of life. (P.36)*

*Seeing how much change we were able to create in a short period of time. Watching the sense of community flourish in the buildings while we were there. (P.49)*

#### Sub-theme 1E: managing participant expectations

Managing the expectations of program participants and trying to elicit health behaviour change was a challenging aspect of the CP role. While seeing positive improvements in participants' lives motivated community paramedics and likely provided them with increased job satisfaction, working with participants who were not able to achieve these positive outcomes in some participants despite working to identify their health issues, and referring and connecting them to services, was a frustrating aspect of the role. Paramedics experienced frustration when participants did not follow their health advice, did not experience improvements in their health, or when participants expressed dissatisfaction with the help they received. Some of this frustration was also directed towards referral agencies who were not able to help the participant.

*Some people are noncompliant with their medications or taking the advice of their physicians. It can be frustrating having people come to you for help for the same problems but not be receptive to the advice that you give. (P. 42)*

*There have been moments of frustration when patients don't follow through or even attempt to follow advice given to them by myself or the agency that has been tasked with giving them assistance. (P. 42)*

*[Some] clients who are out of the normal scope of practice for a paramedic who are better served by other agencies but those agencies failing the client. Even when you help put services in place for a client they are not happy and want more. (P.7)*

#### Sub-theme 1F: interprofessional collaboration

Paramedics enjoyed working with differenthealthcare providers in their community. Collaboration with different services and providers was felt by paramedics to benefit program participants and improve their career satisfaction. Collaboration with different healthcare providers outside of an emergency paramedicine context made paramedics feel respected and part of a valued healthcare team that was centred around improving participant health. This collaboration provided better coordinated care and also showcased paramedics’ clinical skills beyond that of transport and ambulance-driving to other healthcare professions.

*The integration, collaboration, and cooperation with health care and with allied health care providers. We truly make a difference in people's lives, keeping them in their homes longer, safer, and healthier. (P. 67)*

*Building relationships and pathways with community health care providers and showing them that paramedics are more than just ambulance drivers. (P. 13)*

*Interacting with the [primary care provider] as we caught early onset [urinary tract infections (UTIs)] and [upper respiratory tract infections] with treatment started based solely on our assessment and conversation via cell phone with [the provider] saving [the participant] stress and cost of travelling to their office. (P. 49)*

*...Enjoy working more closely with physicians to develop treatment plans.(P.56)*

#### Sub-theme 1G: conflicts with other service providers

While paramedics appreciated the interprofessional collaboration offered by the CP role, they also described conflicts and challenges working with other service providers in the health and social work sector. Paramedics described some service providers as failing and unable to meet participant needs. Overlap between CP activities and other healthcare roles also led to tensions regarding professional boundaries, including physician concerns about CPs diagnosing their patients.

*Some doctors did not like paramedics assessing and diagnosing issues (e.g. chest infections, UTIs, and muscular-skeletal injuries). (P. 39)*

*Don't know if referrals are getting back to [participants]…[There are] already programs in place that have [the] same mandate as CP, like Health Link, forcing medics to do home visits when [participants] don’t need them any more. (P. 12)*

*Oftentimes, navigating the system was a challenge and often wait times with family doctors or other services were unavoidable. (P. 29)*

### Theme 2: CP offers paramedics an opportunity for lateral career movement that is free from the demands of shift work and allows them to be connected to the community in a clinical capacity that is slower paced.

Some respondents viewed CP as a new opportunity for lateral career movement within the paramedic profession, ideal for paramedics in the late-stage of their career as it offered less physically demanding work. It was also noted that CP could help keep aging paramedics in the service for a longer period of time and the community could continue benefiting from their skill set.

After having worked in the new role, paramedics described CP as offering greater freedoms compared to the demands of shift work in traditional emergency response roles. CP offered freedom from the demands of shift work by providing better hours, increased autonomy, reduced physical demands, and reduced paramedic stress. For paramedics with longer years of service, this was a welcomed change of pace, with some reporting mental and physical health improvements. Others noted the importance of still being connected to the community in this new role. For others, adjustment to the slower pace of the CP role was difficult due to their preference for emergency work..

*I enjoyed being still involved with the community but not having to have the daily physical demands of responding to 911 calls. The role is less stressful and after being a paramedic on the road for 14 years it is an amazing and a welcome change of pace both mentally and physically. (P. 58)*

*The autonomy to structure my day without the oversight of dispatch or supervisors. (P.63)*

*[It] would be great for light duty/modified work, could keep aging medics on for [a] longer period of time, good idea for last years of work. (P.51)*

*I prefer a higher paced environment dealing with acute injuries…(P.30)*

### Theme 3: Paramedics viewed and experienced the CP role as a practice paradigm shift

Before working in a CP role, paramedics viewed ed CP to be a practice paradigm shift for the profession. For some, this shift in practice was thought to be in opposition to the traditional emergency care role while others felt it was a natural extension of paramedic practice.

*I did not feel that was something I would enjoy as it does not have the same adrenaline rush you get when on emergency calls. (P. 13)*

*[I] felt it was long overdue and a natural extension of what we were already doing in an emergency capacity. (P. 43)*

*I thought that it would be the next step in emergency medicine, our next frontier. Fire has prevention, we should have health promotion. (P. 26)*

After working in a CP role and experiencing the practice paradigm shift first-hand, paramedics noted being largely satisfied by their newly expanded skill set, but also felt that it was a significant learning curve. Paramedics experienced negative sentiments from their peers in traditional emergency response regarding the CP role, highlighting the diverging paradigms between the two roles.

#### Sub-theme 3A: expanded skill set

The CP program expanded paramedics’ skill set to provide better care to program participants. Some of the new clinical skills described included medication provision, suturing, catheterization, point-of-care testing. Paramedics felt these skills improved their overall ability to perform when returning to emergency response duties. Others felt these new clinical skills were not used or required for the CP role because participants were mainly looking to socialize and interact.

*I very much enjoyed the increased scope of practice. I believe that it allows me to provide better care and assist people in the community more than I have before. Moreover, I feel that the additional training has made me a better, and more well-rounded medic overall. (P.34)*

*I enjoyed the expanded roles (phlebotomy, catheterization, suturing etc)...(P.25).*

#### Sub-theme 3B: learning curve

Working in a CP role was a significant learning curve for some paramedics. Challenges included learning soft skills such as communication, confidence leading sessions with older adults, and learning administrative tasks such as new documentation and computer skills. For paramedics working in both emergency response and CP roles, it was difficult to shift between emergency response protocols and CP protocols. This may have been due to competing priorities between emergency response and CP protocols, such as deciding whether to transport an individual to hospital or keeping an individual at home.

*It is a difficult shift in frame of mind to go from 911 assessments to CP assessments and having to switch back into 911 mode when necessary...It can be tough to play the role of both emerg[ency] response and CP. (P.18)*

*Adapting to new ways, changing the way you do calls, learning the CP documentation and computer programs, being confident with [program participants] and visits, knowing when to communicate with the providers and how. (P. 2)*

*Much more patient advocacy & health teaching then I had expected. (P.14)*

#### Sub-theme 3C: negative paramedic culture

Community paramedics described a negative paramedic culture that is unaccepting of the CP role and its softer skill set. Lack of buy-in from paramedics in traditional emergency response roles, along with poor understanding of the positive impacts of CP programming, have led to negative perceptions of the role in the paramedic workforce. Community paramedics felt that their emergency response colleagues did not respect their role and felt misunderstood by the profession at large.

*Paramedic culture that needs to be educated and changed on the value of CP work. (P.32)*

*Misunderstood by co-workers and some management. Labeled the tea and cookie brigade. (P.24)*

*I also found that EMS crews treated CP with very little mutual respect and understanding... (P. 41)*

## Discussion

There were a number of positive and negative aspects of the CP role identified by paramedic respondents. While the majority of respondents felt that working in a CP program was a largely positive experience, some expressed dissatisfaction and difficulty adapting to the role. Many positive aspects of the CP role also had unintended negative aspects, particularly as it related to paramedics’ sense of professional identity and their mental health experience when working in the CP role. In order to ensure paramedic job satisfaction and understand the future state of CP programs, these opposing experiences need to be further examined and addressed.

### Paramedic professional identity

While many paramedics felt CP was an extension of the paramedic identity, some felt it was a threat to the traditional paramedic identity, removing the defining element of ‘emergency response’ and blurring professional boundaries with other health and social service roles. These diverging experiences and attitudes towards the CP role and its place in the paramedicine profession suggest that there are different fractional identities within the paramedic workforce. Donelley et al. found that emergency service workers often define their role using four domains: caregiving (helping individuals in need), thrill seeking (the adrenaline rush experienced during critical incidents), capacity (having the knowledge, skills, and training to act), and duty (obligation to one’s community and service) [7]. Paramedics who understand their professional identity as falling within the ‘caregiving’ or ‘duty’ domain may be more accepting of the CP role and understand its fit within their existing paramedic mandates. However, paramedics who understand their professional identity as falling within the ‘thrill seeking’ and ‘capacity to conduct an emergency response’ domain may view CP as not only redefining and expanding the profession, but a threat to the professional identity. Expansion and further resourcing of CP programs may exacerbate divisions and tensions between staff who have different professional motivations if these concerns are not addressed.

### Paramedic mental health

Working in a CP role may have also led to some improvements in paramedic mental health. In the traditional emergency response role, paramedics take on shift work, are often exposed to traumatic emergency response incidents, and are limited in their interactions with individuals in their care (single touchpoint and limited time). In contrast, community paramedics experienced more freedom to structure their day, new opportunities to build relationships with program participants due to multiple touchpoints and they experienced reduced physical demands. These experiences likely contributed to a less stressful, flexible work environment which in turn improved mental health for some.

However, increased socialization with participants also introduced new emotional burdens and stressors for some community paramedics. Increased attachment to program participants often made it difficult to deal with their deaths. Participants are often vulnerable populations who face complex health and social issues, such as poverty and addiction. Increased contact with vulnerable populations may increase paramedics’ exposure to vicarious trauma or ‘compassion fatigue,’ which refers to the secondary trauma experienced by working closely with individuals who have experienced trauma first-hand [13–15]. Vicarious trauma and compassion fatigue can have similar negative impacts on paramedic mental health as first-hand trauma, leading to emotional disturbances, stress, intrusive thoughts, and reduced productivity [15]. Particularly for community paramedics with a strong orientation towards empathy and caregiving, compassion fatigue may be experienced as a negative or challenging consequence of the role [15].

### Considerations for CP programming

The experiences of paramedics working in a CP program suggests the CP role comes with new opportunities and challenges for staff and the profession at large. Paramedics have broad and diverse understandings of their professional identity, leading some to view CP as a natural fit within the profession while others view it as extending too far beyond the boundaries of paramedicine. This suggests the need for paramedic leaders to clearly define the purpose, mandate, and function of the CP role within the paramedic workforce. Paramedic services interested in implementing and expanding on CP programs to achieve program outcomes such as a reduction in emergency calls and improving participant health outcomes should reflect on their workplace culture and consider the role of their leadership in promoting this role. Champions of CP programming may be identified to better support the workforce’s understanding of this role and how it fits within larger paramedic mandates and objectives. Paramedic leaders who are championing the CP role should consider what factors may contribute to a paramedic feeling alienated in a CP role and how staff are selected to fill this role. In addition, negative perceptions of the CP role as ‘soft’ or ‘easy’ in comparison to emergency response roles needs to be dispelled if community paramedics are to feel valued for their efforts and contributions.

In addition, a number of training supports may need to be provided that take into consideration the new emotional burdens of the CP role. While the CP role may contribute to good mental health by providing a flexible work environment, reducing exposure to traumatic incidents, and allowing paramedics to socialize with individuals in their care, it may also put some paramedics at risk for vicarious trauma and compassion fatigue. Drawing from professions such as social work and counselling, a number of training and professional development supports can be provided to reduce compassion fatigue. Examining compassion fatigue in community paramedics, Cornelius et al. suggests that paramedics should establish boundaries when working with program participants, ensuring that participants recognize the relationship between them and the paramedic is time limited [15]. Additionally, the caseload of community paramedics should be examined and managed by supervisors in terms of size and complexity of cases [15]. Other paramedic supports could include resiliency training, counselling services, and stress management workshops [15]. Training provided should match the type and scope of the CP program the paramedic is working in and their work environment.

### Limitations

A limitation of this study is that it used an online survey with predefined open-ended questions to extract information on lived experience rather than a semi-structured interview. This approach prevented researchers from prompting paramedics on their responses and engaging in discussion to obtain a deeper description of their experiences. However, the survey approach allowed the research team to obtain responses from a large number of paramedics and collect responses from across Ontario. Another limitation is that due to the inherent nature of the survey link, it cannot be guaranteed that unique responses were captured. However, multiple entries from respondents are unlikely.

Future research should attempt to engage paramedics on the issues described in this paper and should consider how the relative impacts of working in different types of CP programs (e.g., clinic style programs, at home visits, etc.) may affect paramedic experiences. This approach may provide more detailed data to inform future CP training and program design.

## Conclusion

This paper found paramedics who have worked in a CP role, reported that the role offered opportunities to fill a gap in the healthcare system, to move laterally within the paramedic profession, and to create a practice paradigm shift within the profession. Most described having positive perceptions of their professional identity after working as a CP, as they were able to fulfill stepping into a helping role to a greater extent. In contrast, some came out of the experience with negative perceptions. It is important for CP program planners to consider these diverse experiences when planning for the expansion of these programs. A workforce culture that views CP programming negatively and as potentially eroding the traditional paramedic identity may work to hinder the program’s ability to achieve positive outcomes such as a reduction in emergency calls and an improvement in participant health outcomes. Incorporating the CP role within larger paramedic mandates and objectives by paramedic leadership may support this work, as well as CP champions who clarify the role and impacts of CP to staff.

### Supplementary Information

**Supplementary Material 1.** 

## Data Availability

The data that support the findings of this study are not publicly available due to them containing information that could compromise participant privacy. De-identified, limited data will be shared by the corresponding author upon reasonable request.

## Abbreviations

CP: Community Paramedicine
OSI: Occupational Stress Injury
