# Supplementary material for: Paramedic attitudes and experiences working as a community paramedic: a qualitative survey
Source: BMC Emerg Med. 2024 Apr 1;24:50. doi: 10.1186/s12873-024-00972-5 (PMC10983637; doi:10.1186/s12873-024-00972-5)
Supplement: Supplementary file 1 — Supplementary Material 1. [file 12873_2024_972_MOESM1_ESM.pdf]

<u>Supplementary File - Survey Draft</u>

Community Paramedicine Survey

Thank you for accessing our survey on community paramedicine. Researchers from the Department of Family Medicine at McMaster University are inviting you to participate in a study on Community Paramedicine. The goal of the study is to understand the current perceptions and experiences of paramedics with respect to the Community Paramedic role. The survey will inform future Community Paramedicine programs and research. All information provided is anonymous, will be kept confidential, and is for research purposes only. Individual responses will not be shared with your paramedic service.

---

1. Have you ever worked in a community paramedic role (e.g. CHAP-EMS, EPIC, home-visits)?
   ☐ Yes
   ☐ No

The following questions appear if the above answer is yes.

2. What was your opinion of community paramedicine before working a community paramedicine role?
   <open ended text-box>

3. Please explain how your opinion of community paramedicine has changed since working in a community paramedic role?
   <open ended text-box>

4. What was positive about your experience working in a community paramedic role? What did you enjoy about this role?
   <open ended text-box>

5. What were the negative aspects in your experience working as a community paramedic?
   <open ended text-box>

6. Would you like to change anything about the community paramedic role?
   <open ended text-box>

In order to better understand what opinions and voices have been represented, it's important that we know some general information about you:

7. How many years have you been working as a paramedic?
   ☐ 0-4 years
   ☐ 5-9 years

- ☐ 10-14 years
- ☐ 15-19 years
- ☐ 20 or more years

8. What is the highest level of paramedic training received?
   - ☐ Primary Care
   - ☐ Advanced Care
   - ☐ Critical Care
   - ☐ Other, please specify ____________

9. Have you ever been on modified duties?
   - ☐ Yes

     If so, please briefly describe (e.g. injury, pregnancy, only day shifts)? (Text box)
   - ☐ No

10. Sex:
    - ☐ Male
    - ☐ Female
    - ☐ Other

11. Age:
    - ☐ 20-24 years old
    - ☐ 25-29 years old
    - ☐ 30-34 years old
    - ☐ 35-39 years old
    - ☐ 40-44 years old
    - ☐ 45 -49 years old
    - ☐ 50+ years old
